# Dual-specificity mitogen-activated protein kinase kinases can use ADP to phosphorylate MAP kinases *invitro*

**DOI:** 10.1016/j.jbc.2025.110578

**Published:** 2025-08-08

**Authors:** Pauline Juyoux, Jill von Velsen, Erika Pellegrini, Matthew W. Bowler

**Affiliations:** European Molecular Biology Laboratory, Grenoble, France

**Keywords:** kinase, protein kinase, phosphoryl transfer, cell signaling, MAP kinase

## Abstract

Kinases are a diverse group of enzymes that use ATP to phosphorylate a variety of substrates. Protein kinases evolved in eukaryotes as important mediators of cell signaling that target specific amino acid side chains to modulate downstream protein function. Among them, the mitogen-activated protein kinases (MAPKs) are a family of intracellular protein kinases that form signaling cascades responding to a number of stimuli, which control fundamental mechanisms such as proliferation, differentiation, inflammation, and cell death. Signals propagate through consecutive kinases which eventually phosphorylate and activate a MAPK. Here, we show that the dual-specificity threonine/tyrosine mitogen-activated protein kinase kinases (MAP2Ks or MEKs) are able to phosphorylate and activate their substrate MAPKs using ADP as well as ATP *in vitro*. As the pathways are involved in the stress response, we speculate that it would represent an advantage to be able to maintain signaling under conditions such as hypoxia, which occur under a number of cell stresses, including cancer and atherosclerosis, where the available pool of ATP could be depleted.

Phosphorylation is a posttranslational modification that is extensively used in the control of processes in higher organisms (1). Protein kinases are important mediators of cell signaling by targeting specific amino acids for phosphorylation, which modulates downstream protein function. One such family of protein kinases is the mitogen-activated protein kinases (MAPKs) that form signaling cascades, responding to a number of stimuli, which control fundamental mechanisms such as proliferation, differentiation, inflammation, and cell death (2). Signals propagate through kinases which eventually phosphorylate and activate a MAPK, through double phosphorylation at a TxY motif in the activation loop (A-loop) by a specific MAPK kinase (MAP2K). Once activated, the MAPK modulates the expression of genes through activation of transcription factors or other protein kinases (3, 4).

Kinases have evolved to phosphorylate their substrates using ATP as it is the ubiquitous currency for energy transduction in cells and is around 10 to 1000 times more abundant than ADP in the cell (5). ADP-dependent kinases were initially identified in extremophiles (6, 7, 8) and were originally thought to be an adaptation to high temperatures, or be an evolutionary remnant of an ancient metabolic pathway. Subsequently, ADP-dependent kinases have been identified in all kingdoms of life, including mesophiles (9) and vertebrates (10, 11, 12, 13). However, they are rare, cannot use ATP, and are restricted to sugar and cysteine substrates (14). The only kinase so far found to be able to use different nucleotides is the kinase domain of *Dictyostelium* myosin-II heavy chain kinase A, a member of the atypical α-kinases, which have no similarity to eukaryotic protein kinases; this α-kinase is a serine threonine protein kinase that has been shown to be able to phosphorylate substrates using ATP, ADP, and AMP (15). Nucleophilic attack on the β-phosphate of ADP is chemically similar to the γ-phosphate of ATP, with the same standard transformed Gibbs energies of formation in physiological conditions (16). However, most enzymes have evolved to precisely position substrates for nucleophilic attack, meaning they are either ATP- or ADP-dependent. Additionally, the concentration of ATP is usually much higher than ADP making it a more abundant substrate, with the distance from equilibrium driving the many processes that depend on ATP. Many proteins are sensitive to this ratio, such as ATP-dependent potassium channels (17) and the pseudo kinase domain of IRE1 (18), moving from an ATP-bound to an ADP-bound conformation, allowing cells to react to the metabolic state. This is important as there are many conditions that lead to drastic changes in the ATP/ADP ratio, such as hypoxia, which require cells to react. Here, we demonstrate that the human MAP2K dual-specificity protein kinases can use both ATP and ADP to transfer phosphate groups to the A-loop of their target MAPKs *in vitro*.

## Results

### The A-loop of p38α is phosphorylated on residues Thr180 and Tyr182 by MKK6^DD^ in the presence of ADP

**Figure 1:**
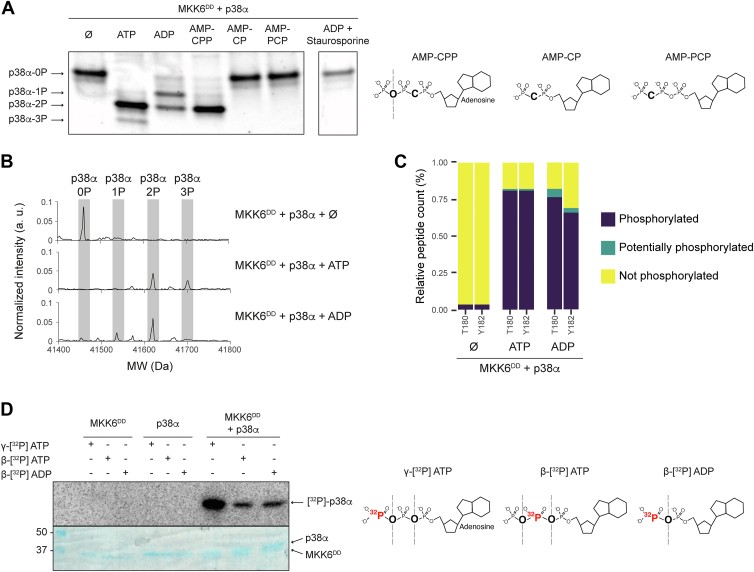
**MKK6 can phosphorylate p38α using ADP *in vitro*.***A*, native PAGE of MKK6^DD^ + p38α in the presence of different nucleotides and an inhibitor. The p38α bands run depending on the number of phosphorylated residues. Chemical structures of the different nucleotide analogs are represented. The *dashed lines* represent bonds that are available for hydrolysis by the kinase. See Fig. S1 for the uncropped gel. *B*, measurement by ESI-QTOF MS of the phosphorylation of p38α by MKK6^DD^. Each phosphorylation increases the mass by 80 Da. *C*, phosphorylation of the p38α A-loop T180 and Y182 residues by MKK6^DD^ measured by LC-MS/MS. Results are represented as the percentage of peptide counts detected as not phosphorylated (*yellow*), potentially phosphorylated (*blue*), and phosphorylated (*purple*) by MASCOT analysis. *D*, *in vitro* phosphorylation assay with radiolabeled nucleotides. Samples of MKK6^DD^ and p38α were incubated with γ-[^32^P] ATP, β-[^32^P] ATP, or β-[^32^P] ADP. Samples were separated by SDS-PAGE and visualized by autoradiography (*top*) and Coomassie stain (*bottom*). Chemical structures of the different radiolabeled nucleotides are represented, with the radiolabeled ^32^P in *red*. A-loop, activation loop; MS, mass spectrometry.

The active conformation of MAP2Ks is stabilized by the double phosphorylation of serine and threonine residues in their A-loops (S207 and T211 in MKK6). We used a constitutively active mutant, named MKK6^DD^, where the mutation to aspartates mimics phosphorylation. While preparing complexes for structural studies (19), we assessed the ability of MKK6^DD^ to phosphorylate p38α in the presence of different nucleotides. Native PAGE gels were used to separate the different phosphorylation states of p38α (Fig. 1*A*) with the bands identified by mass spectrometry (MS). As expected, in the presence of ATP, MKK6^DD^ transfers two phosphate groups to p38α (p38α-2P) (and also, to a lesser degree, a third (p38α-3P)), indicating high activity. We also observed that in the presence of ADP, MKK6^DD^ can phosphorylate p38α, resulting in a mixture of both monophosphorylated (p38α-1P) and biphosphorylated (p38α-2P) p38α. We obtained similar results using MKK6^WT^, which displays basal activity, and using p38α^K53R^ (a p38α kinase-dead mutant) (Fig. S1*A*), ruling out p38α autophosphorylation. We complemented this analysis with native ESI-QTOF MS experiments, as well as LC-MS/MS detection of phosphorylated sites on samples in solution (Fig. 1, *B* and *C*), which confirmed that both the T180 and Y182 residues of the p38α A-loop were phosphorylated by MKK6^DD^ in the presence of either ADP or ATP with no preference for one residue over the other. In order to rule out ATP contamination, we repeated the experiments with ultra-pure ADP, obtaining the same result, and confirmed the absence of ATP in our ADP stocks *via* natural abundance ^31^P NMR (Fig. S2).

### MKK6^DD^ can use the β-phosphate of both ADP and ATP to phosphorylate p38α

Direct proof of the incorporation of the β-phosphate of ADP was obtained with a radionucleotide assay (Fig. 1*D*). Incubation of MKK6^DD^ and p38α in the presence of either β-[^32^P] ADP or β-[^32^P] ATP resulted in the transfer of the labelled phosphate to the substrate. Given that the β-phosphate can be transferred from either ATP or ADP, we initially speculated that the dual phosphorylation could proceed *via* a single step, using first the γ-phosphate of ATP and, subsequently, the β-phosphate of the resulting ADP product in a fully processive mechanism. However, by incubating MKK6^DD^ and p38α with AMP-CPP (adenosine-5'-[(α,β)-methyleno]triphosphate), an ATP analog from which the β-phosphate cannot be cleaved, we demonstrated the use of the β-phosphate is not required to achieve dual phosphorylation (Fig. 1*A*) and ADP phosphorylation is therefore an alternative route to phosphorylation of the A-loop. Additional controls using the noncleavable nucleotide analog AMP-PCP (adenosine-5'-[(β,γ)-methyleno]triphosphate) ruled out the addition of a pyrophosphate to a single residue from the cleavage of the α-β phosphodiester of ATP. In addition, the use of an active site competitive inhibitor, staurosporine, and of the noncleavable ADP analog AMP-CP (adenosine-5'-[(α,β)-methyleno]diphosphate) showed no phosphorylation activity. These controls demonstrate that the transfer of phosphate in the presence of ADP occurs *via* nucleophilic attack of the β-phosphate in the active site of MKK6.

### Phosphorylation with ADP is less efficient than with ATP

**Figure 2:**
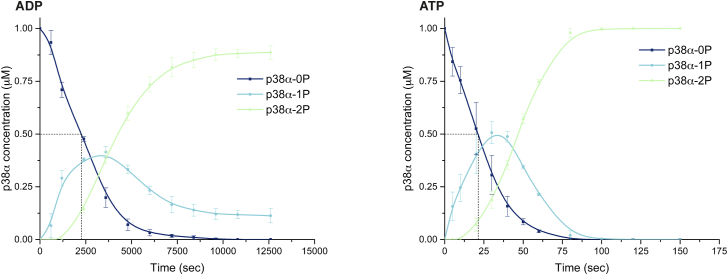
**Phosphorylation using ADP is slower than with ATP.** Time course phosphorylation assay of p38α by MKK6^DD^ with ADP (*left*) and ATP (*right*). The *dashed line* represents the point at which half of the p38α population is phosphorylated at least once, 2243 s (37 min) with ADP, and 21 s with ATP. Concentrations of the different phosphorylation states of p38α were calculated for triplicates of native PAGE gels and fit with a b-spline curve. See Fig. S1 for representative gels.

We then proceeded to a time course phosphorylation assay with either ADP or ATP (Fig. 2) showing that phosphorylation using ADP is ∼100 times slower than with ATP, but the curves are similar between the nucleotides and match previous studies with ATP (20, 21). The point at which half of the p38α is phosphorylated at least once, under the conditions used for our assay, is reached in 21 s with ATP and 2243 s (37 min) with ADP. While this is significantly slower, it is still on a biologically relevant timescale.

The *in vitro* dual phosphorylation of p38α by MKK6^DD^, in the presence of both ATP and ADP, appears to occur through a distributive mechanism, with a pool of monophosphorylated p38α population building up first, and the biphosphorylated population appearing afterward (Fig. 2). This delay in the formation of biphosphorylated p38α was previously observed (20) and is indicative of a distributive mechanism as a single first product is produced quickly before rebinding leads to the second phosphorylation event.

Attempts to perform a complete kinetic study of the system faced limitations. Given the difference in speed between the reaction using ADP or ATP, it was difficult to find experimental conditions (temperature, kinase and substrate concentration, time course) measurable and comparable with both nucleotides. In addition, the dual phosphorylation of the p38α A-loop by MKK6 does not follow classical Michaelis–Menten kinetics. As predicted by simulation studies, and assays with the MEK1-ERK2 system (22), a nonprocessive (or distributive) mechanism predicts that there will be a paradoxical decrease in the rate of MAPK phosphorylation as the MAPK concentration is increased, a phenomenon we also observed.

In some experimental conditions, the dual phosphorylation of p38α with ADP was incomplete during the experimental timeline (Figs. 1*A* and S1). Kinetic studies with ATP have shown that the second phosphorylation reaction occurs more slowly than the first (20, 21). This would be enhanced in the slower reaction with ADP, with the phospho-group of p38α-1P making it more difficult for the second phospho-acceptor to access the ADP, possibly due to steric hindrance.

### All MAP2Ks can use ADP to phosphorylate their MAPK substrates

**Figure 3:**
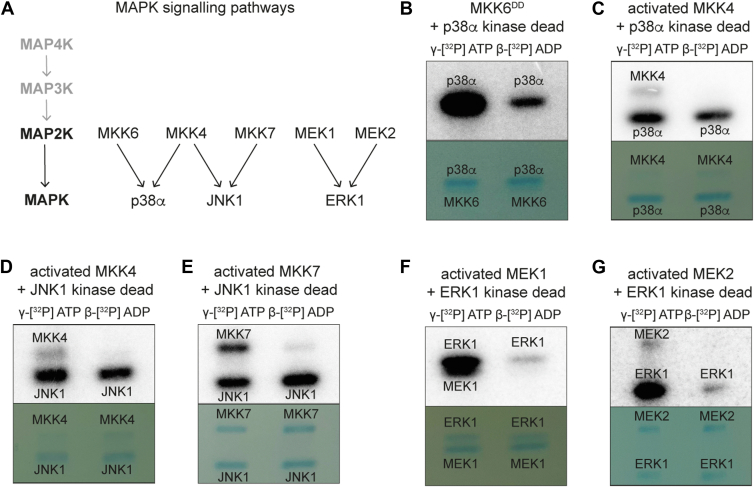
**All MAP2Ks can use ADP to phosphorylate their MAPK substrates**. *A*, schematic showing the specificity of MAP2Ks for MAPKs. *B*–*G*, *in vitro* phosphorylation assay with radiolabeled nucleotides. Pairs of activated MAP2K (MKK6^DD^, MKK4, MKK7, MEK1, and MEK2) and kinase dead MAPK (p38α, JNK1, and ERK1) were incubated with γ-[^32^P] ATP or β-[^32^P] ADP. Samples were separated by SDS-PAGE and visualized by autoradiography (*top*) and Coomassie stain (*bottom*). MAPK, mitogen-activated protein kinase; MAP2K, MAPK kinase.

We then tested other activated MAP2K–MAPK pairs. All the tested MAP2Ks were able to phosphorylate their target MAPKs using either ATP or ADP (Fig. 3), with different relative activities depending on the MAP2K. In comparison to the reduced MKK6 activity with ADP, MKK4 appears to be similarly active on p38α with either ATP or ADP (Fig. 3*C*). MKK4 and MKK7 activity on JNK1 was similar with either ATP or ADP as a phosphate source. MKK7 might even be slightly more active with ADP than with ATP (Fig. 3*E*). The activity of both MEK1 and MEK2 on ERK1 was low with ADP in comparison to ATP, but still occurs. The differences in ADP phosphorylation between MAP2Ks could suggest that it might be an evolutionary advantage in some pathways and not purely an *in vitro* observation.

**Figure 4:**
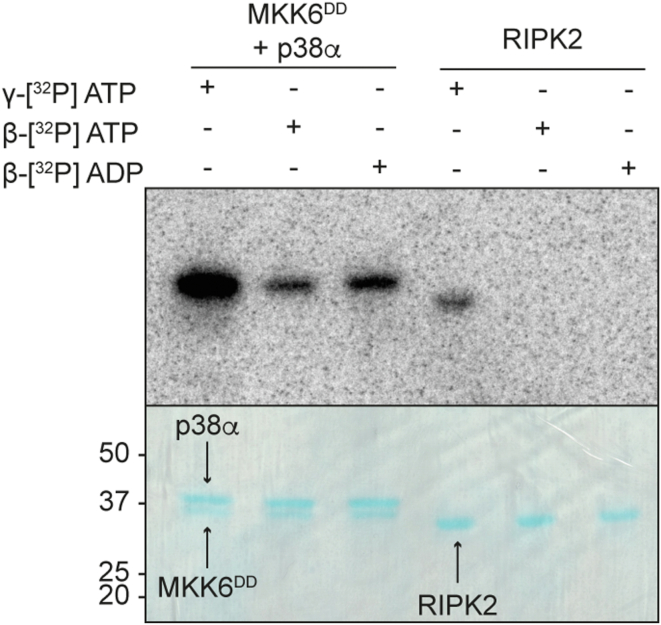
**RIPK2 cannot use ADP to autophosphorylate**. *In vitro* phosphorylation assay with radiolabeled nucleotides. Samples of MKK6^DD^ + p38α and RIPK2 were incubated with γ-[^32^P] ATP, β-[^32^P] ATP, or β-[^32^P] ADP. Samples were separated by SDS-PAGE and visualized by autoradiography (*top*) and Coomassie stain (*bottom*).

As a further control, we also tested the ability of a kinase from a different branch (TLK) of the kinome (23), the kinase domain of RIPK2 (24), to use ADP as a phosphate donor (Fig. 4). RIPK2 activation occurs through autophosphorylation at the A-loop, where up to six closely located phosphorylation sites are present (24, 25). Moreover, during signaling, RIPK2 can phosphorylate a tyrosine residue in the RIPK2 CARD domain (26), showing the ability of this kinase to phosphorylate both serine/threonine and tyrosine residues, unusual in protein kinases, making it similar to MAP2Ks. Our data demonstrate that this kinase is not able to use ADP to autophosphorylate, suggesting the use of ADP as substrate is not a universal mechanism in protein kinases. However, it remains possible that other kinases more closely related to the MAP2Ks could have this ability as well.

## Discussion

A small number of kinases have evolved to position ADP for nucleophilic attack on the β-phosphate. These ADP-dependent kinases were initially discovered in extremophiles where it was thought that high temperatures could lead to depleted levels of ATP. However, subsequent discovery of ADP-dependent kinases in mesophiles, and even in vertebrates, has shown that they are more likely adapted for alternative metabolic routes or sensing the state of the cell (11). These ADP-dependent kinases are rare and have evolved to use ADP specifically and, to date, only an atypical α-kinase has been shown to use both ATP and ADP (15). We have demonstrated that the human dual-specificity protein kinases of the MAPK pathway, the MAP2Ks, are able to phosphorylate and activate their target MAPKs using both ATP and ADP *in vitro*. The incorporation of the β-phosphate occurs both in the presence and absence of ATP but is less efficient than the use of the γ-phosphate and is not essential to activate the target MAPK. This suggests that the use of ADP is an alternative route to MAPK activation, for example, when the concentration of ATP is very low.

MAP2Ks are an unusual family of protein kinases referred as "dual-specificity kinases:" they can phosphorylate two chemically and structurally different amino acids, tyrosine and threonine, separated by a single residue (TxY motif). The majority of protein kinase families phosphorylate either the structurally similar residues serine and threonine or are specific for tyrosine (and in some cases histidine). Recent structural studies from our laboratory have demonstrated that, as opposed to a classical enzyme, where substrates are precisely positioned, the MAP2K MKK6 engages with its substrate MAPK p38α at sites distal from the A-loop (19). This allows flexibility in the amino acid side chain targeted for phosphorylation and could also allow either ATP or ADP to be approached for nucleophilic attack using the same active site architecture. As the β-phosphate is deeper in the active site of the MAP2K, and therefore less accessible to the A-loop, this could account for the lower efficiency observed with ADP phosphorylation of p38α. In addition to the lower efficiency of activation, there is also a lower proportion of doubly phosphorylated p38α. This could allow for alternative signaling, as it has been suggested that monophosphorylated p38α and ERK could have alternative signaling roles (27, 28, 29).

As the MAPK pathways are generally involved in the stress response, it is conceivable that the ability to maintain signaling in a low ATP environment would be an advantage, for instance under hypoxic conditions, which occur under a number of cell stresses, such as cancer and atherosclerosis. It has been clearly demonstrated that MAPK signaling is maintained, and often activated, during these hypoxic conditions (30, 31, 32, 33). Cells are extremely sensitive to the ATP/ADP ratio with many proteins sensitive to the ratio triggering metabolic responses, maintaining stress signaling under these circumstances is also important. The ATP/ADP ratio drops significantly within minutes of ischemia, for example from 10 to 0.4 within 90 s in rat cerebellum (34). The use of ATP in the cell depends on its displacement from equilibrium, which is lost upon the collapse of the ATP/ADP ratio, a problem that could be circumvented by the alternative use of ADP. The observation that the MAP2Ks can use ADP to activate their target MAPKs *in vitro* is surprising and needs to be demonstrated if and when it occurs *in vivo*. The observation also means that kinase kinetic assays based on the formation of ADP should be reassessed. The phenomenon may be limited to the unusual dual-specificity MAP2Ks, but it would seem prudent to test as wide a range of protein kinases as possible in order to determine how limited the ability to use ADP is in protein kinases. This study is an invitation to scientists in the protein kinase field to test their kinase's ability to phosphorylate using ADP.

## Experimental procedures

### Plasmids

Plasmids for protein expression were ordered from GenScript. The sequences for p38α, ERK1, and JNK1 (WT and kinase-dead mutants) were fused to a His6 tag and 3C protease cleavage site, and cloned into pET-28b. MKK6 sequences (WT and constitutively active S207D T211D mutant, “MKK6^DD^”) were fused to a twin StrepII tag and 3C site, and cloned into pFastBac1. Lambda Phosphatase plasmid was ordered from Addgene (#79748) (35).

## Protein expression and purification

p38α, ERK1, and JNK1 constructs were cotransformed with lambda phosphatase plasmid into Rosetta(DE3)pLysS *E. coli* (Novagen). Cells were grown in LB at 37 °C to A_600_ = 0.6 to 0.8, induced with 0.5 mM IPTG, incubated at 16 °C overnight, and harvested. MKK6 constructs were transformed into DH10 *E. coli* to generate baculoviruses for expression in *Sf21* insect cells (36) and harvested 48 h after proliferation arrest by centrifugation.

Pellets were resuspended in lysis buffer (50 mM Hepes pH 7.5, 200 mM NaCl, 10 mM MgCl_2_, 5% glycerol, 0.5 mM TCEP, protease inhibitor, traces of DNaseI), lysed by sonication on ice, and centrifuged.

p38α, ERK1, and JNK1 supernatants were purified using a 5 ml HisTrap column (GE) and equilibrated with wash buffer (same as lysis buffer minus DNaseI) plus 1% elution buffer (500 mM Imidazole). Eluted fractions were pooled, incubated with 3C-protease, dialyzed overnight at 4 °C, and re-run on HisTrap; flow-through collected.

MKK6 supernatant was purified on a 5 ml StrepTactin XT column (IBA), equilibrated in wash buffer. Elution used 50 ml wash buffer with 50 mM biotin. Fractions were pooled, digested with 3C-protease, dialyzed overnight, and re-run on the same column.

The RIPK2 kinase domain was purified as previously described (24). Activated MAP2Ks (MEK1, MKK3, MKK4, MKK7) were purchased from ProQinase; MEK2 from R&D Systems.

## Nucleotides

Nucleotides and analogs were purchased from Merck, ultra-pure ADP from Cell Technology.

## Phosphorylation assays using native PAGE gels

Endpoint assays were prepared on ice in 25 μl with 1 μM MKK6^DD^ and 1 μM p38α in assay buffer. Nucleotides were at 0.5 mM unless stated; reactions incubated at 30 °C for 20 min. For inhibitor assays, 0.5 mM Staurosporine (Merck, 569397) was added prior to nucleotide. Reactions were quenched with native sample buffer (10% glycerol, 20 mM Tris pH 6.8, traces of bromophenol blue, 25 mM EDTA).

Time course assays used 500 μl reactions with 0.2 μM MKK6^DD^ and 1 μM p38α, 1 mM nucleotides. Samples were taken at various time points and quenched. All samples were loaded on precast 4 to 20% Tris-glycine gels (Thermo Fisher Scientific) and run in Tris-glycine native buffer (2.5 mM Tris, 19.2 mM glycine, pH 8.3) at 125 V for 1.5 h. Gels were stained (InstantBlue or Coomassie), imaged using ChemiDoc (Bio-Rad), and analyzed in ImageLab (v6.0, https://www.bio-rad.com/fr-fr/product/image-lab-software?ID=KRE6P5E8Z).

### Mass spectrometry

#### Intact mass by Q-TOF MS

Samples (5 μg) were acidified with 1% TFA and injected onto a UPLC BEH C4 column (Waters) on the Acquity UPLC System, coupled to a Q-TOF Premier MS (Waters/Micromass) using electrospray ionization in positive mode. Solvent A: 0.1% formic acid; solvent B: acetonitrile + 0.1% formic acid. Data were acquired over 500 to 3500 *m/z* in continuum mode with a scan time of 0.5 s. Calibration was performed with myoglobin. Intact mass was calculated using MaxEnt1 (Waters/Micromass). The scores were normalized to the sum of intensities between 41.4 and 41.8 kDa measured for each run and plotted as a function of molecular weight (a. u.).

#### Digestion and posttranslational modifications analysis by LC-MS/MS

Samples were prepared following the SP3 protocol (37). Analysis was performed on an UltiMate 3000 RSLC nano LC system (Dionex) fitted with a trapping cartridge (μ-Precolumn C18 PepMap 100) and an analytical column (nanoEase M/Z HSS T3 column, Waters) coupled directly to a Fusion Lumos (Thermo Fisher Scientific) mass spectrometer using the proxeon nanoflow source in positive ion mode.

Peptides were introduced into the Fusion Lumos *via* a Pico-Tip Emitter (New Objective) and an applied spray voltage of 2.4 kV. Full mass scan was acquired with mass range 375 to 1200 *m/z* in profile mode in the orbitrap with resolution of 120,000. The filling time was set at maximum of 50 ms with a limitation of 4 × 105 ions. Data-dependent acquisition was performed with the resolution of the Orbitrap set to 30,000, with a fill time of 86 ms and a limitation of 2 × 105 ions. A normalized collision energy of 34 was applied. MS2 data were acquired in profile mode. Acquired data were processed by IsobarQuant (38), the Mascot (v2.2.07) search engine was used. For each amino acid position of interest (p38α T180 and Y182), results are represented as the percentage of peptide counts corresponding to each Mascot Delta score (39) category (not phosphorylated, “MD-score < 32” = localization probability of phosphorylation at indicated position < 75%, and “MD score ≥ 32” = localization probability of phosphorylation at indicated position >75%).

### Phosphorylation assays using radiolabeled nucleotides

Proteins at 0.5 μM in 10 μl assay buffer were prepared on ice. Radiolabeled nucleotides (γ-^32^[P] ATP, β-[^32^P] ATP, β-[^32^P] ADP, Hartmann Analytic) were diluted 1:10 into 1 mM cold nucleotide (ATP or ADP). One microliter nucleotide was added per sample and incubated for 20 min at 30 °C. Reactions were stopped with 4 μl SDS sample buffer (0.4% bromophenol blue, 0.4 M DTT, 0.2 M Tris, pH 6.8, 8% SDS, 40% glycerol), boiled 5 min at 95 °C, centrifuged, and loaded on 4 to 20% Tris-glycine gels (Thermo Fisher Scientific), run in Tris-glycine buffer (2.5 mM Tris, 19.2 mM glycine, pH 8.3, 1% SDS) at 220 V for 40 min. Gels were exposed to phosphor screens (GE) overnight, imaged using Typhoon (GE), then stained, and reimaged. Images were analyzed in Image Lab (Bio-Rad).

### ^31^P NMR spectra of nucleotides stocks

Spectra (10 mM ATP/ADP) were acquired on a Bruker 700 MHz spectrometer with cryoprobe at 25 °C and processed in TopSpin. Peaks were assigned by comparison with published work (40).

## Data availability

The mass spectrometry proteomics data have been deposited to the ProteomeXchange Consortium *via* the PRIDE (41) partner repository with the dataset identifier PXD065314.

## Supporting information

This article contains supporting information.

## Conflict of interest

The authors declare that they have no conflicts of interest with the contents of this article.
