# Supplementary material for: Dual-specificity mitogen-activated protein kinase kinases can use ADP to phosphorylate MAP kinases invitro
Source: J Biol Chem. 2025 Aug 8;301(9):110578. doi: 10.1016/j.jbc.2025.110578 (PMC12446509; doi:10.1016/j.jbc.2025.110578)
Supplement: Supplementary Data [file mmc1.docx]

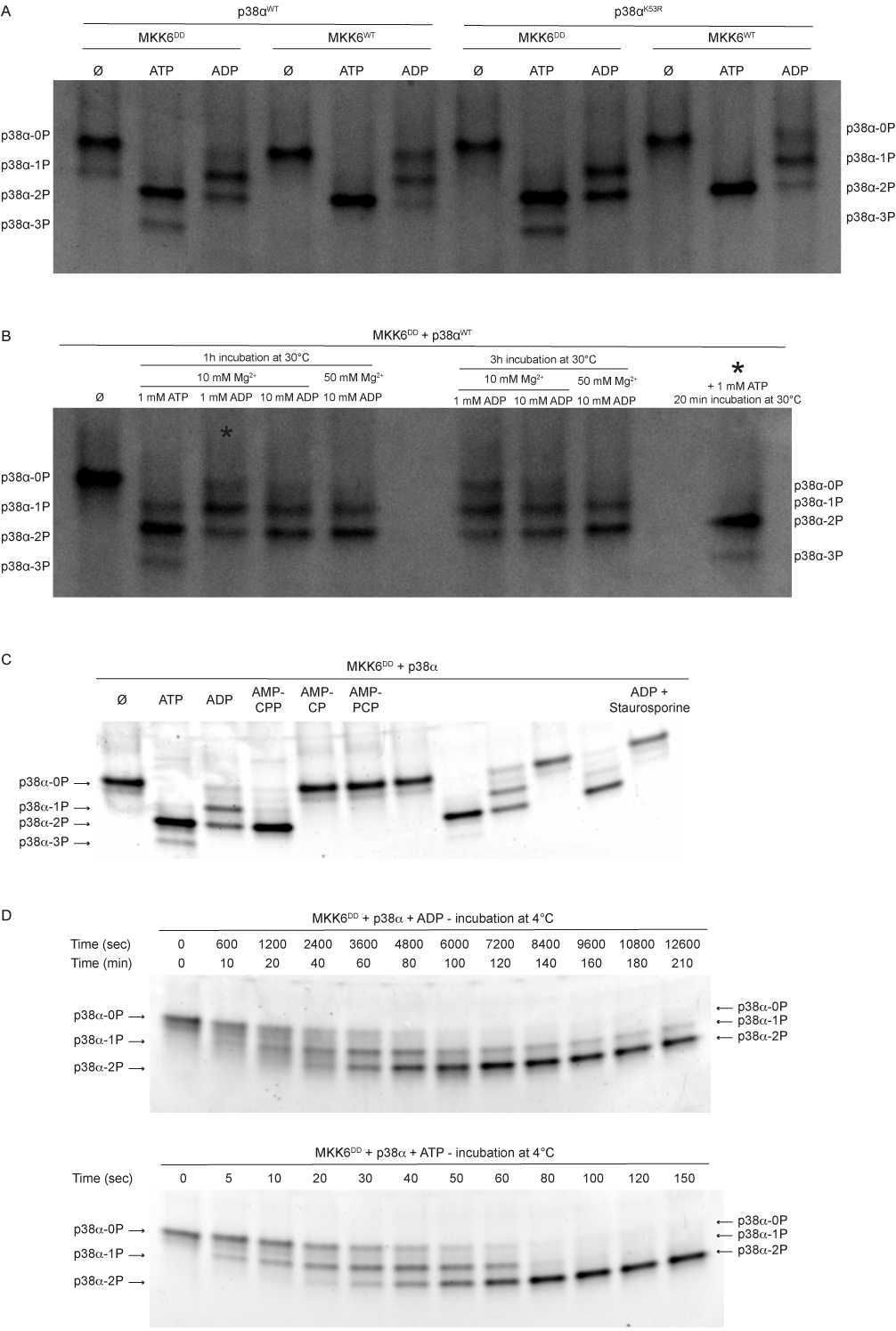


**Figure S1. ADP phosphorylation of p38α variants by MKK6 variants under different conditions**

(A) Native PAGE gel of MKK6 (WT or constitutively active DD mutant) + p38α (WT or kinase-dead K53R mutant) in the presence of nucleotide. p38α bands run based on the total phosphorylation number of the protein.

(B) Native PAGE gel of MKK6^DD^ + p38α^WT^ in the presence of different nucleotides and Mg^2+^ concentrations, and longer incubation time. The last sample was initially incubated for 1h at 30°C with 1 mM ADP, and was then supplemented with 1 mM ATP and incubated for 20 additional minutes before being run on the gel. p38α bands run based on the total phosphorylation number of the protein.

(C) Uncropped gel from Fig 1A.

(D) Representative gels used to generate Fig 2.


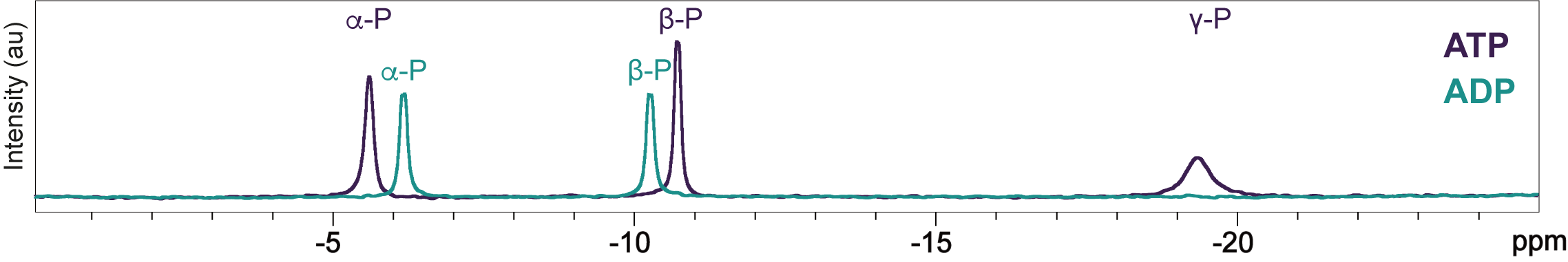


**Figure S2. Nucleotide purity**

Natural abundance ^31^P NMR spectra of ATP and ADP stock solutions used in *in vitro* assays demonstrating the absence of ATP in the ADP solutions.
